# Extracellular vesicles in cancer: golden goose or Trojan horse

**DOI:** 10.1093/jmcb/mjae025

**Published:** 2024-05-25

**Authors:** Tao Han, Qian Hao, Tengfei Chao, Qinggang Sun, Yitian Chen, Bo Gao, Liping Guan, Wenjie Ren, Xiang Zhou

**Affiliations:** Institutes of Health Central Plains, Xinxiang Key Laboratory for Molecular Oncology, Xinxiang Medical University, Xinxiang 453003, China; Fudan University Shanghai Cancer Center and Institutes of Biomedical Sciences, Fudan University, Shanghai 200032, China; Department of Oncology, Shanghai Medical College, Fudan University, Shanghai 200032, China; Department of Oncology, Tongji Hospital, Tongji Medical College, Huazhong University of Science and Technology, Wuhan 430030, China; Institutes of Health Central Plains, Xinxiang Key Laboratory for Molecular Oncology, Xinxiang Medical University, Xinxiang 453003, China; School of Basic Medical Sciences, Xinxiang Medical University, Xinxiang 453003, China; Institutes of Health Central Plains, Xinxiang Key Laboratory for Molecular Oncology, Xinxiang Medical University, Xinxiang 453003, China; School of Basic Medical Sciences, Xinxiang Medical University, Xinxiang 453003, China; Umibio Co. Ltd, Shanghai 201210, China; School of Basic Medical Sciences, Xinxiang Medical University, Xinxiang 453003, China; Institutes of Health Central Plains, Xinxiang Key Laboratory for Molecular Oncology, Xinxiang Medical University, Xinxiang 453003, China; Fudan University Shanghai Cancer Center and Institutes of Biomedical Sciences, Fudan University, Shanghai 200032, China; Department of Oncology, Shanghai Medical College, Fudan University, Shanghai 200032, China; Key Laboratory of Breast Cancer in Shanghai, Fudan University Shanghai Cancer Center, Fudan University, Shanghai 200032, China; Shanghai Key Laboratory of Medical Epigenetics, International Co-laboratory of Medical Epigenetics and Metabolism (Ministry of Science and Technology), Institutes of Biomedical Sciences, Fudan University, Shanghai 200032, China

**Keywords:** exosome, tumor microenvironment, immune response, metastasis, cancer therapy

## Abstract

Intercellular communication can be mediated by direct cell-to-cell contact and indirect interactions through secretion of soluble chemokines, cytokines, and growth factors. Extracellular vesicles (EVs) have emerged as important mediators of cell-to-cell and cell-to-environment communications. EVs from tumor cells, immune cells, and stromal cells can remodel the tumor microenvironment and promote cancer cell survival, proliferation, metastasis, immune evasion, and therapeutic resistance. Most importantly, EVs as natural nanoparticles can be manipulated to serve as a potent delivery system for targeted cancer therapy. EVs can be engineered or modified to improve their ability to target tumors and deliver therapeutic substances, such as chemotherapeutic drugs, nucleic acids, and proteins, for the treatment of cancer. This review provides an overview of the biogenesis and recycling of EVs, discusses their roles in cancer development, and highlights their potential as a delivery system for targeted cancer therapy.

## Introduction

Intercellular communication is critical for cancer development. Such activity can be facilitated by the exchange of extracellular vesicles (EVs), which contain nucleic acids, proteins, and metabolites, from donor cells to recipient cells. The secretion of EVs was initially considered a waste disposal pathway that eliminates unneeded cellular components. It was later realized that EVs are more than waste carriers and play essential roles in transferring components between cells (Couch et al., 2021). EV-mediated cell-to-cell communication is associated with cancer initiation, progression, and immune response (van Niel et al., 2018; Dixson et al., 2023). Several oncogenic proteins, such as MYC, AURKB, and mutant p53, have been found to educate a tumor-promoting microenvironment through the secretion of EVs (Cooks et al., 2018; Novo et al., 2018; Kilinc et al., 2021), whereas tumor suppressors foster a tumor-suppressive microenvironment via EVs (Schuldner et al., 2019; Zhang et al., 2020b). Because of the biocompatibility of EVs and their ability to deliver cargos, EVs have been exploited to develop new targeted therapeutics for cancer. In this review, we summarize recent progress on the functions and mechanisms of EVs in cancer development and, particularly, emphasize the role of EVs in the development of novel anticancer therapy.

## Life cycle of EVs

**Figure 1:**
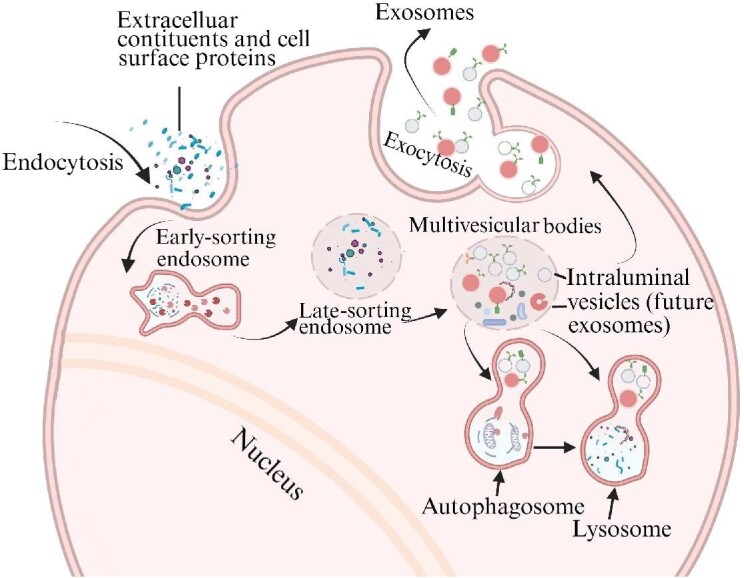
Biogenesis and release of exosomes. Unlike ectosomes, exosomes arise from double invagination of the plasma membrane.

EVs generally fall into two major categories, exosomes and ectosomes (or microvesicles), based on their distinct biogenesis pathways (Kalluri and LeBleu, 2020). Of note, the term ‘exosome’ has also been widely used to describe the RNA-processing machinery (Houseley et al., 2006). While ectosomes in the size range of 50–1000 nm in diameter are generated by direct outward budding at the plasma membrane, exosomes that are generally in a size range of 30–150 nm in diameter arise from double invagination of the plasma membrane (Figure 1). Endocytosis, the first invagination of the plasma membrane, leads to the formation of early-sorting endosome containing extracellular constituents and cell surface proteins. Early-sorting endosomes can mature into late-sorting endosomes, followed by a second invagination. The inward budding of the endosomal membrane gives rise to multivesicular bodies (MVBs) that contain intraluminal vesicles. MVBs can be transported to the plasma membrane through the cytoskeletal and microtubule network. Intraluminal vesicles are ultimately released to the extracellular space as exosomes through exocytosis. Alternatively, MVBs can also be fused with autophagosomes or lysosomes for degradation. Numerous molecules have been found to be critical for the biogenesis of EVs, such as the endosomal sorting complexes required for transport (ESCRT) family proteins (Hurley, 2008; Colombo et al., 2013), Ras-related protein GTPase Rab (Hsu et al., 2010; Wang et al., 2014), ADP-ribosylation factor 6 (Muralidharan-Chari et al., 2009), apoptosis-linked gene 2-interacting protein X (Baietti et al., 2012), soluble *N*-ethylmaleimide-sensitive factor attachment protein receptor complex proteins (Jahn and Scheller, 2006; Fader et al., 2009), etc. However, the detailed mechanisms for the biogenesis and secretion of EVs are still being revealed.

**Figure 2:**
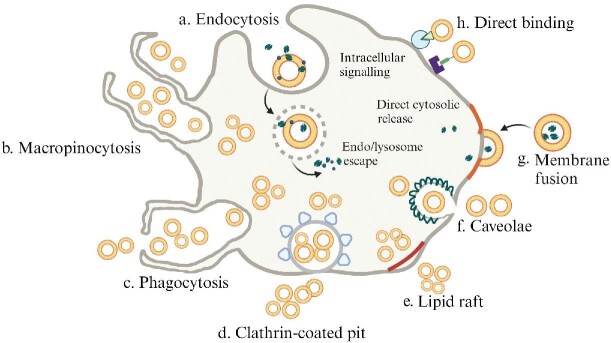
Cellular uptake of EVs. EVs bind to recipient cell surface to trigger various intracellular signaling pathways or be internalized through different mechanisms.

Once secreted into the extracellular space, EVs can reach and bind to recipient cell surface. The bound EVs may remain at the plasma membrane to trigger various intracellular signaling pathways or can be internalized through different manners (Figure 2; Mulcahy et al., 2014). The internalization can be executed through receptor-mediated endocytosis (Nechaev et al., 2013; Gonda et al., 2019), macropinocytosis (Tian et al., 2014a; Li et al., 2020), phagocytosis (Feng et al., 2010), clathrin-coated pits (Tian et al., 2014a; Li et al., 2020), lipid rafts (Sapon et al., 2023), or caveolae (Le Saux et al., 2020), resulting in the entry of intact EVs, which are contained in MVBs. These EV-containing MVBs are usually targeted to lysosomes, which leads to the degradation of their carried proteins and lipids, thereby providing a relevant source of metabolites to the recipient cells. In some cases, these internalized EVs may escape from lysosomal degradation via back-fusion with the MVB membrane, releasing their contents into the cytoplasm, or via re-secretion into the extracellular space. In addition, EVs can directly fuse with the plasma membrane to deposit their contents into the cytoplasm of recipient cells, which bypasses lysosomal surveillance and is essential for cell uptake of therapeutic cargoes that are delivered by EVs.

## Roles of EVs in cancer

**Figure 3:**
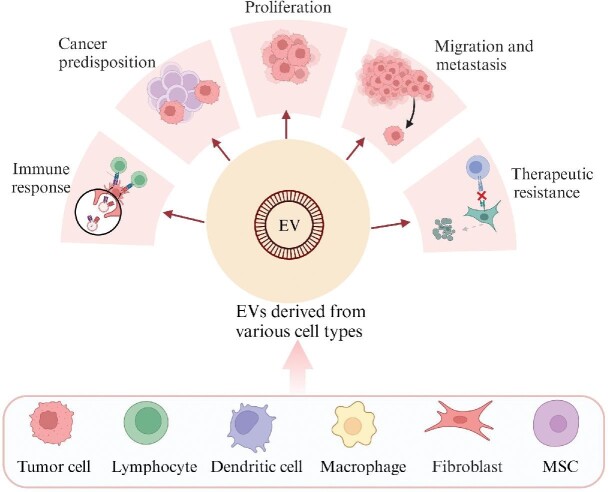
Roles of EVs in cancer. EVs mediate cancer predisposition, proliferation, migration and metastasis, immune response, and therapeutic resistance.

The crosstalk between cancer cells and the tumor microenvironment is critical for tumor growth, metastasis, and immune evasion. The tumor microenvironment includes a large variety of cell types, such as endothelial cells, fibroblasts, lymphocytes, and macrophages, as well as the extracellular matrix (ECM). EVs that carry various nucleic acids, proteins, lipids, and other bioactive molecules have been found to be involved in multiple aspects of cancer development and therapeutic resistance by remodeling the tumor microenvironment (Figure 3; Kalluri and McAndrews, 2023; Sohal and Kasinski, 2023).

The rapid growth of tumors requires the activation of proliferative signals and inhibition of growth suppressors (Hanahan and Weinberg, 2011). EV-mediated signal or cargo transfer plays an important role in these biological processes. For instance, EVs derived from fibroblasts, endothelial cells, and granulocytes contain c-Myc, miR-126, and miR-320, respectively, foster a tumor-favoring microenvironment, thereby increasing the predisposition to lung cancer (Pontis et al., 2021). In addition, cancer cell-derived EVs carrying c-Myc, ZIP4, or an active form of epidermal growth factor receptor (EGFR) promote their own proliferation in an autocrine manner (Al-Nedawi et al., 2008; Jin et al., 2018; Borzi et al., 2019). EVs also regulate the migration and distant metastasis of tumor cells (Becker et al., 2016). EVs containing proteolytic enzymes, such as matrix metalloproteinases and ADAM (a disintegrin and metalloprotease domain) family proteins, change the ECM compositions (Shimoda and Khokha, 2017). Moreover, cancer-associated fibroblasts, which are the most abundant stromal cells in the tumor microenvironment, secret EVs that can remodel the ECM to facilitate the spread of tumor cells (Li et al., 2021). Importantly, EVs can facilitate pre-metastatic niche formation and promote the metastasis of tumor cells to distant organs. The seminal study by Hoshino et al. (2015) reported that EVs from lung-, liver-, and brain-tropic tumor cells are preferentially taken up by lung fibroblasts and epithelial cells, liver Kupffer cells, and brain endothelial cells, respectively. They also showed that organotropic tumor exosomes create pre-metastatic environments for the metastasis of tumor cells that are not typically expected to colonize those areas (Hoshino et al., 2015). One of the mechanisms is through the transfer of exosomal integrins to resident cells. These integrins induce Src phosphorylation and pro-inflammatory *S100* gene expression, which is required for pre-metastatic niche formation (Hoshino et al., 2015). Additional cargoes that mediate EV-induced cancer cell metastasis include multiple miRNAs, lipids, and proteins as reviewed elsewhere (Becker et al., 2016; Hu et al., 2023; Kalluri and McAndrews, 2023; Sohal and Kasinski, 2023).

EVs also regulate immune activation or suppression to control cancer initiation and progression (Marar et al., 2021). In 1996, EVs were discovered to act as immunomodulators. Raposo et al. (1996) found that both human and murine B lymphocytes release EVs that are enriched with major histocompatibility complex (MHC) class II to trigger T cell response. A later study reported that mature dendritic cells (DCs) release antigen-presenting EVs that contain MHC class I, MHC class II, and T cell costimulatory molecules, which activate cytotoxic T cell response to eradicate tumor cells (Zitvogel et al., 1998). As a major type of antigen-presenting cells, DCs produce EVs that can induce maturation of various types of immune cells (Wei et al., 2017; Schierer et al., 2018), orchestrate differentiation of type 1 helper T (Th1), Th2, and regulatory T cells (Cai et al., 2012; Tkach et al., 2017), and promote activation of CD4^+^ and CD8^+^ T cells (Lindenbergh et al., 2019; Cui et al., 2020; Hagerbrand et al., 2022). Conversely, activated T cells transfer EVs that contain genomic and mitochondrial DNAs to DCs, thereby inducing antiviral response and conferring protection to DCs (Torralba et al., 2018). Myeloid-derived suppressor cells can transfer GPR84 via EVs to induce senescence of CD8^+^ T cells by activating the p53 pathway (Liu et al., 2023). Tumor cells also release EVs to modulate immune response to influence their own growth. For instance, tumor EVs that carry PD-L1 on the membrane can directly activate PD-1/PD-L1 immune checkpoint, thus impairing the proliferation of CD8^+^ T cells, inhibiting interleukin 2 (IL-2) and interferon gamma (IFNγ) production, and increasing overall tumor burden (Chen et al., 2018; Ricklefs et al., 2018; Kim et al., 2019; Daassi et al., 2020). Depletion of EV-associated PD-L1 suppresses tumor growth and overcomes tumor resistance to anti-PD-L1 antibody therapy (Poggio et al., 2019). Besides, tumor EVs can deliver oncogenic mutant p53 to induce a tumor-promoting microenvironment and suppress CD4^+^ T lymphocytes (Dong et al., 2023). Interestingly, cancer cell-derived EVs can also stimulate antitumor immune response. They deliver tumor-associated antigens and peptide–MHC complexes for antigen presentation (Dionisi et al., 2018; Lin et al., 2020) or DNA fragments to induce maturation in DCs via the cyclic GMP–AMP synthase–stimulator of IFN genes (STING) pathway (Kitai et al., 2017).

Moreover, EVs can induce tumor resistance to therapies (Yang et al., 2022). EVs from tumor cells transfer functional cargoes that mediate resistance from resistant cells to sensitive cells, thereby promoting drug export (Ifergan et al., 2005; Safaei et al., 2005) or modulating cell survival signals that negate the cytotoxic effect of drugs (Maacha et al., 2019). For instance, cisplatin-resistant gastric cancer cells can release EVs that contain miR-769-5p, leading to the inactivation of p53 and cisplatin resistance in sensitive cells (Jing et al., 2022). Also, tumor-supporting cells, like cancer-associated fibroblasts, release EVs containing Annexin A6, miR-21, or lncRNA H19, which contributes to the development of stem-like properties and resistance to chemotherapy in tumor cells (Au Yeung et al., 2016; Ren et al., 2018; Uchihara et al., 2020).

## Surface engineering for enhanced cancer targetability

As natural lipid nanoparticles, EVs have advantages over conventional lipid nanoparticles. EVs display higher biocompatibility and lower immunogenicity, as they do not carry exogenous factors that are recognized and targeted by the immune system. For the same reason, they themselves are less cytotoxic as drug carriers. Another merit of EVs is that they can cross the blood–brain barrier (BBB), thus providing an excellent delivery strategy to treat brain tumors, such as glioma (Rufino-Ramos et al., 2017; Macedo-Pereira et al., 2023). In addition, EVs may exhibit specificity for targeting tumors. For example, mesenchymal stem cell (MSC)-derived EVs can be recruited to primary and metastatic tumors (Weng et al., 2021; Hu et al., 2023; Meng et al., 2023), while tumor cell-derived EVs show some targeting specificity to the parental tumors (Kalluri and McAndrews, 2023). Furthermore, EVs from MSCs or DCs can modulate the tumor microenvironment by transferring immunomodulatory factors (Nail et al., 2023; Rebelo et al., 2023). In recent years, an increasing number of clinical trials have been conducted to evaluate the safety and effectiveness of EV-based cancer therapies (Table 1).

**Table 1:** Clinical trials of EV-based cancer therapies.

Name	Institute	Cancer	Stage	NCT number
iExosomes	M.D. Anderson Cancer Center	Pancreatic cancer with KRAS-G12D mutation	Phase 1	NCT03608631
exoSTING	Codiak BioSciences	Advanced solid tumors	Phase 1/2	NCT04592484
exoASO-STAT6	Codiak BioSciences	Advanced HCC and liver metastases	Phase 1	NCT05375604
DC-derived exosomes	Gustave Roussy, Cancer Campus	Non-small cell lung cancer	Phase 2	NCT01159288
Chimeric exosomal tumor vaccines	Fudan University Pudong Hospital	Recurrent or metastatic bladder cancer	Phase 1	NCT05559177
Plant exosomes	University of Louisville	Colon cancer	Phase 1	NCT01294072
Plant exosomes	University of Louisville	Head and neck cancer with oral mucositis	Phase 1	NCT01668849
UCMSC-Exo	Wuhan Union Hospital	Myelosuppression in acute myeloid leukemia	Phase 1	NCT06245746

**Figure 4:**
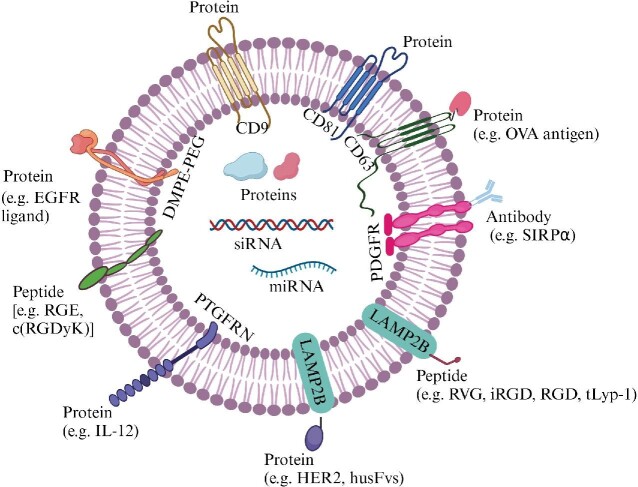
Engineering EVs for cancer therapy. EVs can be engineered with chemicals, nucleic acids, peptides, and proteins.

EVs can be engineered or modified to improve their targeting specificity for tumors (Liang et al., 2021). Generally, the strategies include genetic and chemical modifications (Figure 4). Genetic engineering involves the fusion of tumor-targeting or tumor-penetrating peptides with transmembrane proteins, including LAMP2B, PDGFR, PTGFRN, CD63, CD9, and CD81, on EVs and the expression of the fused protein in donor cells, which produce EVs with the functional peptides on the surface. The *LAMP2* gene encodes three protein isoforms, LAMP2A, LAMP2B, and LAMP2C. While LAMP2A is required for chaperone-mediated autophagy and LAMP2C may play a role in the degradation of RNA and DNA (Eskelinen et al., 2002, 2006), LAMP2B is enriched on the surface of EVs and thus considered a bridge connecting EVs to peptides with different properties. LAMP2B comprises an N-terminal 29-amino acid signal peptide, a large extracellular domain, and a C-terminal transmembrane domain, followed by a very short cytoplasmic domain. Thus, tumor-targeting peptides, such as RGD (Ruoslahti, 1996), RVG (El-Andaloussi et al., 2012), and tLyp-1 (Bai et al., 2020), are usually fused to the N-terminus of LAMP2B. For example, EVs expressing LAMP2B fused with the αv integrin-specific RGD peptide, which homes to tumors by binding to αv integrins (Ruoslahti, 1996), can specifically deliver anticancer drugs to tumors. Hepatocellular carcinoma (HCC)-derived EVs that express RGD peptides on the surface were found to have profound potential in the treatment of HCC (Wu et al., 2021). The modification of EVs using RGD also enhances the therapeutic effect of EV-delivered paclitaxel in the treatment of pancreatic cancer (Al Faruque et al., 2022). RGD-modified EVs loaded with siRNAs against PD-L1, which recruits tumor-associated myeloid cells, can coordinate with radiation to suppress the growth of glioblastoma (Tian et al., 2022). Moreover, RGD-modified EVs have shown prominent tumor targetability in the treatment of ovarian cancer, gastric cancer, osteosarcoma, etc. (Du et al., 2022; Guo et al., 2022; Zhao et al., 2022). Internalizing RGD (iRGD), an improved RGD peptide, was found to home to tumors by binding to both αv integrins and neuropilin-1 (Sugahara et al., 2009). It was reported that iRGD-modified EVs produced by mouse immature DCs and loaded with doxorubicin via electroporation can specifically inhibit αv integrin-positive breast cancer cell growth *in vitro* and *in vivo* (Tian et al., 2014b). iRGD-modified EVs from HEK-293T cells were also shown to deliver doxorubicin combined with radioactive ^131^I to target αv integrin-overexpressing anaplastic thyroid carcinoma (Wang et al., 2022). In addition, iRGD-tagged EVs containing microRNA antagonists efficiently suppress the angiogenesis and growth of nasopharyngeal carcinoma by upregulating the tumor suppressor SPRY3 (Wang et al., 2020). Furthermore, iRGD can facilitate the infiltration of lymphocytes by binding to neuropilin-1, which overcomes the endothelial barrier, to improve immunotherapy (Ding et al., 2019), thus making this tumor-penetrating peptide more fascinating in EV-based therapies.

Besides, targeting proteins or antibodies can be genetically displayed on the surface of EVs (Liang et al., 2021). Human epidermal growth factor receptor 2 (HER2) can activate multiple intracellular pathways to support tumor cell growth and proliferation. The HER2-encoding gene *ERBB2* has been found to be amplified or overexpressed in various cancers, including breast, gastric, colorectal, esophageal, lung, bladder, endometrial, and ovarian cancers (Galogre et al., 2023), which makes this membrane-bound protein an effective target for cancer therapy (Baselga and Swain, 2009). Recently, EVs decorated with the HER2–LAMP2B fusion proteins were reported to efficiently home to HER2-positive colon cancer (Liang et al., 2020). In addition, a humanized anti-HER2 single-chain variable fragment (husFvs) that can specifically recognize HER2 epitopes was constructed (Ou-Yang et al., 2018). The husFvs fused with LAMP2B was displayed on the surface of EVs, which efficiently deliver GSDMD-N mRNAs to HER2-positive breast cancer cells, consequently inducing tumor pyroptosis and immune response (Xing et al., 2023).

EV surface can be chemically modified to increase cancer targetability. Chemical reactions are responsible for the conjugation of peptides or antibodies to EV surface (Liang et al., 2021). For example, the neuropilin-1-targeted peptide RGE was conjugated to EVs by a cycloaddition reaction of sulfonyl azide (Jia et al., 2018). The peptide c (RGDyK) was conjugated to EV surface using bio-orthogonal copper-free azide alkyne cyclo-addition (Tian et al., 2018). Both modifications increased the ability of EVs to cross the BBB. Modification with a polyethylene glycol (PEG) corona is another decoration to increase the compatibility and solubility while reducing the immunogenicity of nanoparticles, such as EVs. EVs, which were decorated with ligands specific for EGFR, conjugated to phospholipid (DMPE)-PEG were reported to target EGFR-overexpressing tumor cells with increased cell specificity and prolonged circulation time (Kooijmans et al., 2016). This is consistent with another study showing that incorporation of EVs with aminoethyl anisamide-PEG improves the circulation time in the blood and the targetability to pulmonary metastases (Kim et al., 2018).

## Cargo loading for cancer therapy

EVs are suitable for the delivery of various antitumor drugs, as hydrophobic molecules can be inserted in their lipid bilayer and hydrophilic molecules can be loaded in their lumen (Figure 4; Choi et al., 2022; Liu et al., 2022). The therapeutic cargoes can be divided into three categories: chemotherapeutic drugs, nucleic acids, and proteins. Chemotherapeutic drugs include a broad spectrum of molecules that can eradicate cancer cells by perturbing cell cycle, impairing microtubule stability, triggering DNA damage, or targeting the growth-promoting signaling pathways. Engineered EVs have been shown to efficiently deliver paclitaxel (Saari et al., 2015; Al Faruque et al., 2022), 5-fluorouracil (Liang et al., 2020), doxorubicin (Jang et al., 2013; Tian et al., 2014b), celastrol (Song et al., 2021), curcumin (Zhuang et al., 2011), sorafenib (Zhang et al., 2020a), imatinib (Bellavia et al., 2017), and senaparib (Chen et al., 2022).

Nucleic acids, such as mRNAs, miRNAs, and siRNAs, which modulate gene expression, can be delivered by EVs for cancer therapy (Zhang et al., 2022). The first attempt to deliver therapeutic mRNAs for cancer treatment was made in 2018. Wang et al. (2018) reported that EV-based HchrR6 mRNA delivery leads to the activation of the prodrug CNOB, efficiently inhibiting the growth of HER2-positive breast cancer cells. GSDMD-N mRNA, as aforementioned, was delivered by EVs to recipient cells to induce pyroptosis and immune response (Xing et al., 2023). In another study, human red blood cell-derived EVs were shown to transport Cas9 mRNAs to edit the genomes in both human cells and xenograft mouse models (Usman et al., 2018). Moreover, EVs may serve as a vector for the development of a variety of mRNA vaccines (Tsai et al., 2021; Popowski et al., 2022; Zhang et al., 2022).

Small inhibitory RNAs, such as miRNAs, siRNAs, and antisense oligonucleotides (ASOs), are a group of promising drugs for targeted cancer therapy. For example, GE11 peptide-modified EVs deliver let-7a miRNA to EGFR-expressing xenograft breast tumors, inhibiting the expression of HMGA2 mRNA and thus the tumor development *in vivo* (Ohno et al., 2013). Apo-A1-modified EVs loaded with miR-26a suppress the growth of HepG2 cells by regulating the expression of cell cycle-associated genes (Liang et al., 2018). miRNA inhibitor-containing EVs were also described to overcome chemoresistance. For instance, miR-21 was shown to trigger chemoresistance in multiple cancers (Giovannetti et al., 2010; Bourguignon et al., 2012; Naro et al., 2018). EVs harboring the miR-21 inhibitor significantly increase tumor sensitivity to doxorubicin (Zhan et al., 2020) and 5-fluorouracil (Liang et al., 2020). Engineered EVs that harbor siRNAs become a powerful strategy for cancer therapy, as this approach can efficiently target previously ‘undruggable’ genes (Friedrich and Aigner, 2022; Zhang and Zhang, 2023). EVs derived from mesenchymal cells were engineered to deliver siRNAs against oncogenic Kras^G12D^, a driving gene mutation in multiple cancers, leading to the repression of pancreatic cancer in several mouse models (Kamerkar et al., 2017). Han et al. (2021) also reported that engineered EVs loaded with siRNAs specific for SIRT6 suppress the growth and metastasis of SIRT6-driven prostate cancer. Survivin siRNA-containing EVs were shown to suppress the growth of prostate, breast, and colorectal cancers, using different mouse models (Pi et al., 2018). Recently, EVs loaded with siRNAs were shown to boost immunotherapy by targeting PD-L1, CD38, or YTHDF1 in glioblastoma (Tian et al., 2022), HCC (Deng and Ke, 2023), or gastric cancer (You et al., 2023), respectively.

EVs can also be engineered to deliver peptide or protein drugs to malignant sites. It was reported that SIRPα can be displayed on EV surface by fusing with PDGFR, and the engineered EVs block CD47, a ‘don't eat me’ signal, to promote the engulfment of tumor cells by macrophages (Koh et al., 2017). IL-12 was expressed on EV surface by conjugating with PTGFRN, and these EVs were shown to suppress tumor growth by increasing tumor antigen-specific CD8^+^ T cells (Lewis et al., 2021). EVs can also directly deliver antigens to induce T cell response. For instance, the OVA antigen was fused with the EV surface protein CD63. These engineered EVs, as a type of therapeutic vaccine, primed naive mice to induce OVA-specific CD4^+^ and CD8^+^ T cells, resulting in the regression of xenograft tumors (Kanuma et al., 2017).

## Concluding remarks

EVs are a group of important mediators between cell-to-cell communications. By remodeling the tumor microenvironment, EVs from tumor cells, immune cells, and stromal cells can promote cancer cell survival, proliferation, metastasis, immune evasion, and therapeutic resistance. Most importantly, EVs as natural nanoparticles can be manipulated to serve as a potent delivery system for targeted cancer therapy. While tremendous progress has been made in the understanding of the biological functions and potential clinical uses of EVs, numerous unanswered questions remain after decades of research in this field. Although several molecules and complexes, such as the ESCRT and Rab family proteins, have been characterized as essential for EV biogenesis, specific mechanisms underlying EV formation and, particularly, the internalization and lysosomal degradation of EVs in different cell types are still unclear. The internalization of EVs mediated by receptor-mediated endocytosis, macropinocytosis, phagocytosis, clathrin-coated pits, lipid rafts, and caveolae can lead to the formation of MVBs that usually undergo degradation through lysosomes. This may severely impair the uptake and functions of therapeutic cargoes delivered by engineered EVs. Enhancing the ability of EVs to directly fuse with the plasma membrane or preventing lysosome-mediated degradation of MVBs would significantly improve the effectiveness of cargo delivery. In addition, the diverse origins and complex composition of EVs may lead to concerns about their biosafety when used in clinical settings. Finally, the most alluring question is how we can train EVs to specifically target cancer cells in a more precise manner. Conventional approaches like phage display, when coupled with artificial intelligence-driven screening, could expedite the discovery of molecules and peptides that specifically target tumors.

## Notes

**Author contributions:** T.H., Q.H., and T.C. drafted the paper; Q.S. and Y.C. collected materials; B.G. provided important instructions; L.G. drew the figures under the supervision of T.H. and X.Z.; and W.R. and X.Z. supervised the project and revised the paper.
